# Macrophage-centered immune-fibrotic persistence in endometriosis and adenomyosis: non-hormonal immunopharmacological targets

**DOI:** 10.3389/fimmu.2026.1925057

**Published:** 2026-09-21

**Authors:** Chan Yu, Danmiao Sun, An Li

**Affiliations:** 1 Department of Women’s Health and Rehabilitation, Huadu District Maternal and Child Health Hospital, Guangzhou, Guangdong, China; 2 Department of Gynecology, The First Affiliated Hospital of Guangzhou University of Chinese Medicine, Guangzhou, Guangdong, China; 3 Department of Traditional Chinese Medicine, Baiyun District Jiahe Street Community Health Service Center, Guangzhou, Guangdong, China

**Keywords:** adenomyosis, endometriosis, immunopharmacology, inflammasome, macrophage, reproductive immunology

## Abstract

Endometriosis and adenomyosis are commonly managed through endocrine suppression, analgesia and surgery, yet these approaches do not fully explain persistent pain, recurrence, fibrotic remodeling or variable treatment response. A growing body of immune and single-cell evidence supports a complementary view in which hormone-permissive tissue injury licenses macrophage-centered inflammatory, fibrotic and neuroimmune programs. This mini review reframes both disorders as related, but not identical, immune-fibrotic conditions and identifies tractable immunopharmacological nodes. We discuss macrophage plasticity, monocyte recruitment, cytokine and chemokine amplification, inflammasome signaling, TGF-β-driven fibrosis, oxidative and metabolic stress, neuroimmune pain signaling and targeted delivery. We argue that non-hormonal therapy should not be developed simply as an alternative to hormonal suppression. Instead, it should be tested as a phenotype-guided strategy that matches dominant immune-fibrotic programs to mechanism-based interventions while preserving reproductive safety. This framework highlights the need for biomarker-informed patient selection, compartment-specific endpoints and early attention to fertility-compatible translational design.

## Introduction

Endometriosis and adenomyosis differ anatomically, but both create chronic, relapsing gynecological disease rather than isolated anatomical findings. Endometriosis commonly presents with dysmenorrhea, chronic pelvic pain, subfertility and recurrent lesion-associated symptoms, whereas adenomyosis is more closely linked to abnormal uterine bleeding, uterine enlargement, dysmenorrhea and reproductive difficulty (1, 2). Endometriosis is increasingly viewed as a chronic systemic condition, while adenomyosis has moved from a post-hysterectomy histological diagnosis toward an imaging-defined and symptom-linked uterine disorder (1, 3). Because many patients experience symptoms across years of reproductive life, both disorders expose patients to repeated courses of analgesia, hormonal suppression, surgery or assisted reproduction.

Current care is still organized mainly around symptom control, endocrine suppression, lesion removal and fertility planning. Guidelines support individualized use of hormonal therapy, analgesia, surgery and assisted reproduction, and recent trials show that modern endocrine agents can reduce pain in selected patients (4, 5). These options remain central to clinical practice, but they do not fully explain why pain may persist despite limited visible disease, why fibrotic lesions can be refractory, or why recurrence and treatment intolerance remain common clinical problems (6, 7). The therapeutic gap is therefore not simply a need for stronger endocrine suppression, but a need to understand the tissue programs that remain active within lesions, eutopic endometrium and adenomyotic myometrium.

Immune and stromal biology provides one way to define those programs more precisely. Estrogen signaling and progesterone resistance can create a permissive tissue environment, whereas persistent pathology is actively driven by macrophage recruitment and polarization, cytokine and chemokine amplification, oxidative stress, inflammasome activation, neuroangiogenesis and extracellular-matrix remodeling (8–10). Single-cell, spatial and translational studies further suggest that peritoneal lesions, ovarian endometrioma, deep infiltrating disease and adenomyotic myometrium should not be treated as immunologically interchangeable compartments (11, 12). This distinction matters therapeutically: an inflammatory macrophage-rich lesion, a fibrotic nodule and a pain-dominant neuroimmune phenotype may require different target classes and different endpoints (7, 13).

Recent reviews have addressed endometriosis-associated macrophages or the broader endometrial immune microenvironment (14–17). In contrast, this mini review jointly considers endometriosis and adenomyosis through a macrophage-centered framework and uses phenotype-guided non-hormonal immunopharmacology to connect compartment-specific immune-fibrotic programs with therapeutic opportunities across both disorders. We focus on mechanisms that are biologically recurrent and pharmacologically tractable, including macrophage-centered crosstalk, cytokine and chemokine amplification, inflammasome activation, fibrotic remodeling, neuroimmune signaling and biomarker-guided translation. This scope intentionally excludes a full survey of hormonal therapy, surgery, assisted reproduction and complementary interventions, which are clinically important but outside the central immunology question. The aim is to position non-hormonal therapy as a phenotype-guided complement to established gynecological care, with target selection, patient stratification and endpoints matched to the dominant immune-fibrotic program.

## Macrophage-centered immune remodeling

Macrophages are attractive organizing nodes because they link innate immune activation, lesion survival, angiogenesis, fibrosis and pain (**Figure 1**). The endometrial immune environment in endometriosis is altered across lesion and eutopic compartments, and adenomyosis is also associated with systematic immune-cell changes (11, 12, 18). Recent single-cell and spatial studies further show that ectopic lesions contain structured cellular niches rather than a uniform inflammatory infiltrate (19, 20). These observations argue against reducing macrophages to a binary M1/M2 axis. Lesion-associated macrophages occupy plastic states shaped by stromal metabolites, cytokines, extracellular vesicles and local tissue architecture.

**Figure 1:**
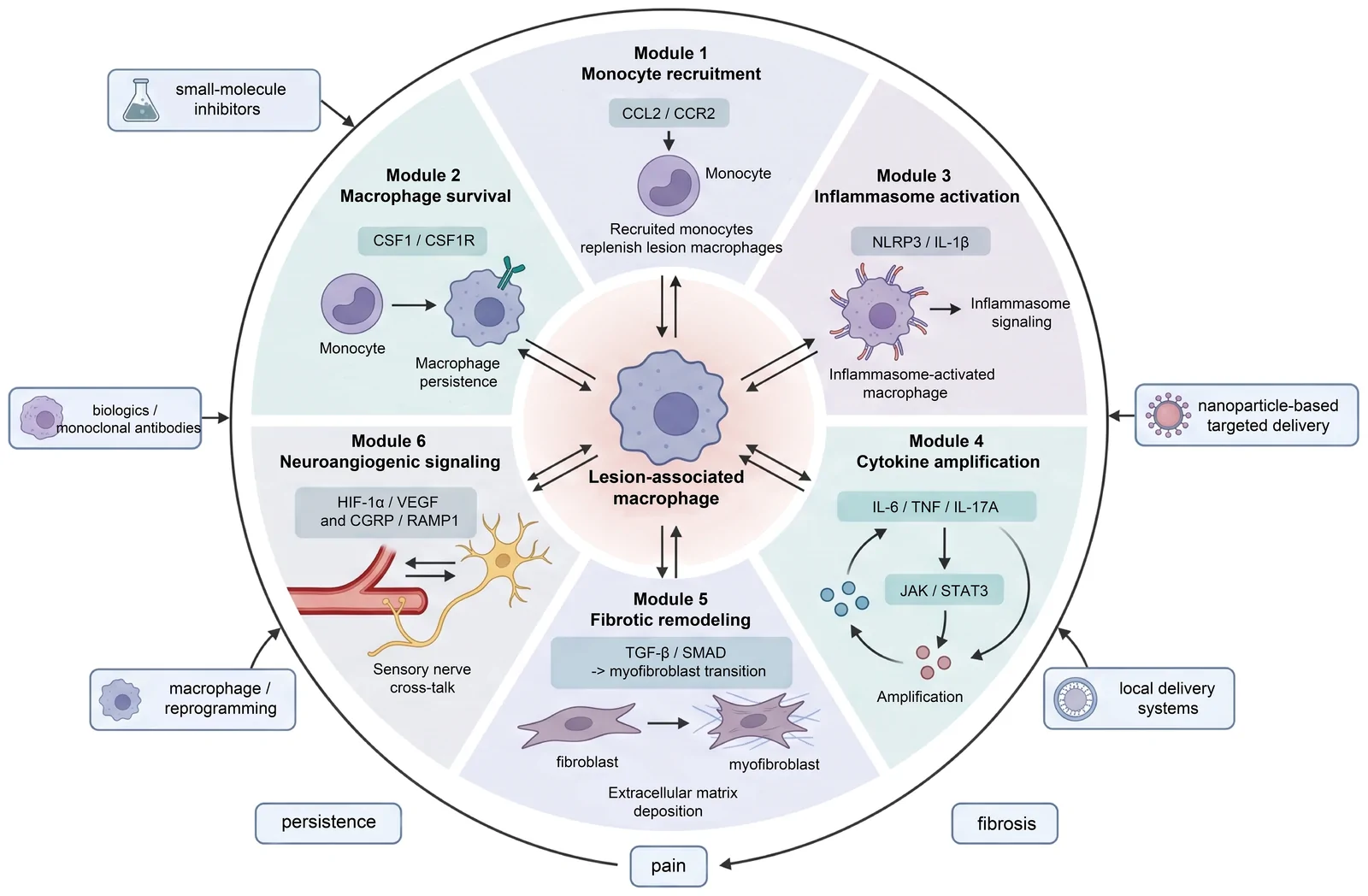
Macrophage-centered immune-fibrotic networks and immunopharmacological strategies in endometriosis and adenomyosis. Lesion-associated macrophages act as central coordinating nodes that drive disease progression through six targetable biological modules: (1) Monocyte recruitment via the CCL2/CCR2 axis; (2) Macrophage survival and persistence driven by CSF1/CSF1R signaling; (3) Inflammasome activation mediated by NLRP3 and IL-1β; (4) Cytokine amplification networks involving IL-6, TNF, IL-17A, and JAK/STAT3 cascades; (5) Fibrotic remodeling characterized by TGF-β/SMAD-induced fibroblast-to-myofibroblast transition and extracellular matrix deposition; and (6) Neuroangiogenic signaling driven by HIF-1α/VEGF and sensory nerve cross-talk via the CGRP/RAMP1 axis. The outer elements highlight how these integrated mechanisms contribute to core clinical phenotypes (persistence, fibrosis, and pain) and outline potential therapeutic interventions, ranging from small-molecule inhibitors and biologics to macrophage reprogramming and advanced nanoparticle-based or local delivery systems.

Mechanistic studies support this view. In endometriosis, stromal-cell-derived lactate can promote macrophage polarization through METTL3/TRIB1/ERK/STAT3 signaling, and macrophages with elevated *TET3* expression have been linked to disease progression (21, 22). In adenomyosis, macrophage polarization has been connected to epithelial-mesenchymal transition and extracellular-vesicle communication (23, 24). These findings make macrophage state, recruitment and survival plausible therapeutic entry points, but they also create a translational warning: broad macrophage depletion may be less desirable than context-specific reprogramming or local interruption of pathogenic crosstalk.

Several macrophage functions are especially relevant for drug development. First, recruited monocytes may replenish lesion macrophage pools and sustain cytokine production (**Figure 1**) (17, 25). Second, macrophages can promote angiogenesis and matrix remodeling, thereby converting transient inflammation into durable lesion architecture (11, 22). Third, macrophages communicate with sensory nerves and stromal cells, linking immune activation to pain and tissue stiffness (**Figure 1**) (13, 26). These functions suggest that a useful macrophage target is unlikely to be a single surface marker. More plausible strategies include interrupting recruitment axes, blocking pathogenic survival signals, shifting macrophage state, or delivering drugs into macrophage-rich lesion niches. Such approaches are especially attractive because they could preserve systemic immune competence while modifying the local lesion microenvironment and limiting unnecessary exposure outside affected tissues.

A further implication is that macrophage-targeted therapy should preserve beneficial immune functions. Macrophages also participate in debris clearance, vascular remodeling, tissue repair and endometrial homeostasis (11). Therapies that indiscriminately suppress macrophages may therefore create unacceptable trade-offs in reproductive-age patients. More competitive strategies are likely to focus on disease-associated signaling states, including chemokine-driven recruitment and inflammasome activation (25, 27), CSF1R-linked survival, pro-fibrotic macrophage-stromal crosstalk and metabolic programs that stabilize pathogenic macrophage phenotypes (20, 21, 28).

Complement adds a further macrophage-linked layer to lesion remodeling. Peritoneal-fluid studies indicate altered regulation of complement pathways in endometriosis, and C1q is increased around lesion blood vessels; lesion CD68+ macrophages are a likely local source of C1q (29, 30). C1q can promote macrophage efferocytosis of apoptotic cells and thereby contribute to tissue remodeling (31), whereas complement activation generates C3a and C5a, anaphylatoxins that recruit and activate leukocytes (32). These context-dependent effects make local complement activity a plausible determinant of lesion persistence and a candidate for mechanistically targeted study.

## Cytokine, chemokine and inflammasome circuits

Cytokine and chemokine networks provide the amplification layer between injured tissue and persistent immune remodeling. The C-C motif chemokine ligand 2 (CCL2)/CCR2 axis is especially relevant because it connects monocyte recruitment to stromal inflammatory signaling in endometriosis and overlaps with older evidence of monocyte chemoattractant protein-1 (MCP-1; also CCL2) and C-X-C motif chemokine ligand 8 (CXCL8; also known as interleukin-8 [IL-8]) expression in adenomyosis (25, 33). IL-1β, IL-6, TNF, IL-17A, CXCL8 and prostaglandin-associated signals further support lesion survival, nociceptive signaling and stromal activation. Rather than cataloguing every mediator, the key priority is identifying actionable immune circuits that can be paired with biomarkers and lesion phenotypes.

The NLRP3 inflammasome is a strong example of such a circuit (**Figure 1**). NLRP3-activated macrophages can promote endometrial stromal-cell migration, and experimental NLRP3 inhibition has been proposed as a non-hormonal strategy for ovarian endometriosis (27, 34). Upstream purinergic and oxidative-stress pathways may converge on similar inflammasome-dependent programs (35). Therapeutically, this suggests a path from innate immune activation to druggable nodes, but reproductive safety, host defense and disease-compartment specificity must be evaluated before systemic innate immune inhibition is advanced clinically.

The same logic applies to JAK/STAT, NF-κB and prostaglandin pathways (**Figure 1**) (36, 37). These cascades are not disease-specific, but their local configuration may be disease-informative when measured with lesion type, menstrual phase, treatment history and symptom phenotype. A key translational gap is that many studies report single mediators without determining whether they define a treatable immune state. Future studies should pair mediator measurements with spatial localization, macrophage-state markers, fibrosis readouts and clinical endpoints (20, 37). Such designs would make it easier to distinguish biomarkers that merely accompany disease from biomarkers that identify a targetable inflammatory program (25, 33, 36).

This distinction is important because cytokine abundance alone does not prove therapeutic vulnerability. A mediator may be elevated because it initiates pathology, reflects secondary tissue injury, or participates in repair. Competitive translational studies should therefore ask whether blocking a candidate pathway changes macrophage state, stromal activation, matrix remodeling, angiogenic support or nociceptive signaling (27, 34, 38). Nociceptive signaling should be assessed with pain-relevant immune-neural models rather than inferred from inflammatory markers alone (13). Such studies would strengthen causal inference and avoid the common problem of treating inflammatory markers as therapeutic targets before their functional role is established (35, 39, 40).

## Fibrosis and neuroimmune persistence

Fibrosis is one of the main reasons that immune targeting should be considered disease-modifying rather than simply anti-inflammatory. Fibrotic extracellular-matrix deposition, myofibroblast differentiation and smooth-muscle-like remodeling contribute to tissue stiffness and treatment-resistant lesion architecture, especially in deep infiltrating disease and adenomyotic myometrium (7, 41). TGF-β/SMAD signaling is a central candidate axis, and recent work has linked TGF-β1/SMAD3-driven macrophage-myofibroblast transition to fibrosis progression in endometriosis (**Figure 1**) (28). The therapeutic implication is that inflammation and fibrosis should be treated as interacting programs rather than sequential and separable events.

Pain adds another immune dimension. Nociceptor-to-macrophage communication through CGRP/RAMP1 signaling can drive endometriosis-associated pain and lesion growth in mice, and macrophage-nerve interactions have long been proposed as contributors to pain biology (**Figure 1**) (13, 26). Oxidative and metabolic programs may further shape this environment; stromal ferroptosis has been linked to angiogenesis, and ferroptosis targeting is emerging as a broader theme in female reproductive disorders (42, 43). These findings support a model in which immune, fibrotic, vascular and neural processes form a self-reinforcing niche.

This niche concept helps explain why symptom improvement and lesion modification may diverge. Endocrine suppression can reduce cyclic stimulation, but established extracellular matrix, nerve ingrowth and inflammatory memory may persist. Conversely, a therapy that reduces cytokine activity may not reverse dense fibrosis unless matrix remodeling or myofibroblast programs are also addressed (7, 28, 41). For Mini Review purposes, the practical message is that immunopharmacology should be aligned with dominant disease programs: inflammatory lesions may require different targets and endpoints from fibrotic nodules, ovarian endometrioma or diffuse adenomyosis (**Figure 2**) (13, 26, 44).

**Figure 2:**
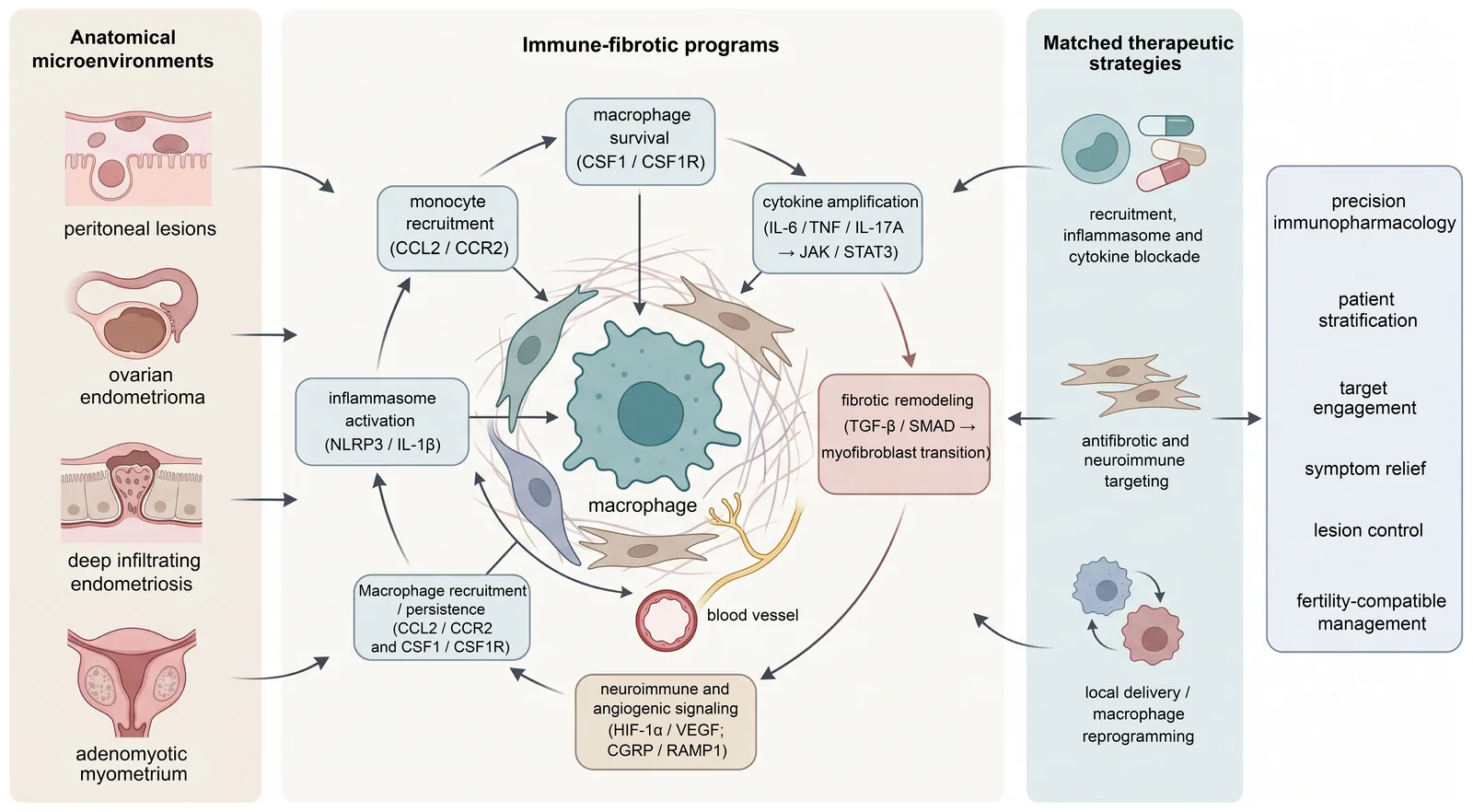
A phenotype-guided translational framework for immunopharmacology in endometriosis and adenomyosis. The framework integrates diverse anatomical microenvironments (left) with core macrophage-centered immune-fibrotic programs (center) to guide matched therapeutic strategies (right). Distinct disease compartments—including peritoneal lesions, ovarian endometrioma, deep infiltrating endometriosis, and adenomyotic myometrium—host shared but context-dependent pathogenic networks. These networks are driven by monocyte recruitment (CCL2/CCR2), macrophage survival (CSF1/CSF1R), cytokine amplification (IL-6, TNF, IL-17A and JAK/STAT3), inflammasome activation (NLRP3/IL-1β), fibrotic remodeling (TGF-β/SMAD), and neuroangiogenic signaling (HIF-1α/VEGF, CGRP/RAMP1). Aligning these specific immune-fibrotic programs with targeted interventions enables precision immunopharmacology, facilitating optimal patient stratification, measurable target engagement, symptom relief, and fertility-compatible clinical management.

## From immune mechanisms to therapeutic hypotheses

For immunopharmacology, the most useful product of mechanism research is not a longer list of candidate targets, but a set of therapeutic hypotheses that can be tested. One hypothesis is that blocking monocyte recruitment will reduce the maintenance of inflammatory lesions (25, 33). A second is that inflammasome inhibition will be most effective in lesions with active IL-1β-linked stromal migration and oxidative stress (27, 34, 35). A third is that anti-fibrotic strategies will require enrichment for matrix-rich or myofibroblast-dominant disease (7, 28). These hypotheses differ in mechanism, target population, biomarker readout and expected clinical endpoint (44).

This target-to-phenotype alignment is important because endometriosis and adenomyosis are not sampled or treated in the same way (**Figure 2**). Peritoneal lesions are exposed to peritoneal fluid, immune-cell trafficking and retrograde-menstruation-linked iron stress (1, 11). Ovarian endometrioma adds ovarian reserve and follicular microenvironment concerns. Deep infiltrating disease often presents with dense fibrosis and nerve involvement (7, 13), whereas adenomyosis is embedded within contractile myometrium and is judged through bleeding, uterine volume, dysmenorrhea and fertility goals (18). The same pathway may therefore require different formulations, treatment windows and proof-of-mechanism endpoints (16, 44).

The field is also not equally mature across targets. Many proposed agents remain supported mainly by animal models, ex vivo assays, retrospective biomarker studies or pathway inference. These data are valuable, but they do not yet establish clinical efficacy or reproductive safety (39, 40). The near-term priority should be disciplined translation: select the immune program, define the patient phenotype, choose a measurable target-engagement marker and match the endpoint to the intended mechanism. This discipline would make non-hormonal therapy a development strategy rather than a broad aspiration (44, 45).

## Therapeutic implications

A non-hormonal immunopharmacological framework should complement, not replace, endocrine suppression. Candidate strategies include modulation of COX-2/PGE2 and pro-resolving lipid pathways, chemokine-axis targeting, inflammasome inhibition, IL-6/JAK/STAT3 and TNF/IL-17 pathway modulation, CSF1/CSF1R-directed approaches, TGF-β/SMAD antifibrotic strategies, VEGF or hypoxia-directed interventions, oxidative-stress control and neuroimmune targeting. Existing reviews of emerging drug targets and pathway-directed pharmacology already support the plausibility of this landscape (39, 40). The next step is not simply adding more targets, but prioritizing those linked to measurable immune-fibrotic phenotypes.

Delivery strategy may determine whether immune targeting becomes clinically usable. Local or lesion-directed delivery could reduce systemic immunosuppression while increasing exposure within pathological niches. Examples include targeted liposomal delivery and immune-cell hitchhiking approaches in experimental endometriosis models (46, 47). For a reproductive-age population, however, translational development must address ovarian reserve, implantation, pregnancy compatibility, systemic immune effects and treatment windows. These constraints should be incorporated into early preclinical design rather than added only after efficacy signals appear.

Combination strategies may ultimately be more realistic than single-agent immune therapy. For example, endocrine suppression could reduce cyclic lesion activation while a non-hormonal agent targets macrophage recruitment, inflammasome activity or fibrotic remodeling (25, 27, 28). Postoperative settings may provide another window in which immune-fibrotic targeting could reduce recurrence or prevent re-establishment of pathogenic niches (6). These concepts remain largely preclinical, but they provide concrete trial questions: which phenotype should be enrolled, what biomarker should change, which symptom or imaging endpoint should improve, and how should reproductive safety be monitored (4, 9, 44)?

Drug repurposing offers a practical entry point, but it should be used selectively. Agents with existing human safety data may accelerate translation, yet a repurposed drug is only persuasive when its known pharmacology matches a defined lesion program (39, 40). For example, an anti-inflammatory compound should be linked to a measurable cytokine or inflammasome phenotype, and an antifibrotic candidate should be tested against matrix and myofibroblast readouts rather than pain alone (28, 34). This requirement also applies to biologics and targeted delivery systems. Without a target-engagement plan, even a plausible pathway can become a generic therapeutic claim. With one, immunopharmacology can be evaluated in a stepwise manner, from mechanism, to biomarker response, to symptom or imaging benefit (40, 46, 47).

## Biomarker-guided translation

The most realistic route forward is phenotype-guided rather than one-size-fits-all therapy (**Figure 2**). Inflammatory-dominant disease may be defined by cytokine, chemokine or inflammasome signatures; fibrotic-dominant disease by imaging features, matrix signatures or myofibroblast-associated programs; pain-dominant disease by neuroimmune markers; and fertility-prioritizing disease by reproductive immune and ovarian reserve constraints. Multi-omics and precision-medicine frameworks already point in this direction (44, 45). The challenge is to convert molecular signals into trial-ready enrichment criteria and clinically meaningful endpoints.

Emerging single-cell and spatial resources may help define these states with more anatomical precision. Integrated endometrial atlases, spatially resolved ovarian endometrioma data and adenomyosis spatial transcriptomic landscapes provide a basis for mapping immune-fibrotic niches across eutopic endometrium, peritoneal lesions, ovarian endometrioma, deep disease and adenomyotic myometrium (48–50). The main conceptual shift is from disease labels to spatially organized immune programs that can be targeted, monitored and combined with established gynecological care (**Figure 2**) (16).

Trial design should therefore move beyond generic pain or lesion volume outcomes alone. Pain scores, bleeding outcomes, imaging of adenomyotic or fibrotic compartments, inflammatory mediator panels, macrophage-rich lesion signatures, quality-of-life measures and fertility-related endpoints may need to be combined according to treatment intent. A macrophage-recruitment inhibitor, an antifibrotic agent and a neuroimmune therapy should not be judged by identical short-term endpoints (7, 13, 25). The field will advance faster if early studies specify the immune program being targeted and select the patient subgroup most likely to express that program (16, 44, 45).

## Outstanding questions

Several questions should be prioritized before non-hormonal immunopharmacology can move from conceptual promise to clinical testing. Which macrophage states are reproducibly pathogenic across lesion compartments, and which are protective or required for tissue repair? Are cytokine and inflammasome signatures stable enough to guide treatment, or do they vary too strongly with cycle phase, prior hormonal exposure and lesion sampling site (11, 20)? Can fibrotic and neuroimmune phenotypes be defined with non-invasive imaging, circulating markers or minimally invasive fluid biomarkers? These questions matter because immune-targeted therapy will be difficult to justify if patients cannot be selected on the basis of a measurable target program (7, 17, 23).

A second set of questions concerns therapeutic boundaries. Many candidate pathways, including IL-6/JAK/STAT3, NLRP3, CSF1R, TGF-β/SMAD and VEGF, are important in normal immunity, tissue repair, ovarian physiology and pregnancy. This does not preclude targeting them, but it argues for local delivery, time-limited treatment, compartment-specific endpoints and careful reproductive toxicology (16, 40). The field also needs clearer criteria for success. A drug intended to reduce inflammatory pain may not need to shrink lesions, whereas an antifibrotic strategy should be expected to alter stiffness, imaging features or matrix-associated biomarkers (7, 13). Matching mechanism to endpoint will be essential for credible clinical translation (28, 44).

A final question is how to compare immune-targeted therapy with existing care. The goal should not be to replace hormonal or surgical treatment in all patients, but to define settings in which immune-fibrotic targeting adds a benefit that current approaches do not provide (4, 5). Such settings may include recurrence prevention, refractory pain, fibrotic disease and fertility-aware management (6, 7, 13).

## Conclusion

Endometriosis and adenomyosis should be viewed as hormone-permissive disorders in which macrophage-centered immune-fibrotic circuits contribute to persistence, pain and treatment resistance. The strongest therapeutic opportunities cluster around macrophage state and recruitment, cytokine and inflammasome amplification, TGF-β-linked fibrosis, neuroimmune signaling, metabolic stress and targeted delivery. The major limitation of the current field is not a shortage of candidate targets, but insufficient linkage between targets, patient phenotypes, reproductive safety and trial endpoints. A phenotype-guided immunopharmacology strategy may therefore provide the most credible path toward non-hormonal, fertility-compatible disease modification. The immediate task is to make this path experimentally specific: define the immune lesion state, select the matching target, measure target engagement and choose endpoints that reflect the biology being modified. If that discipline is applied, immune-directed treatment could evolve from a collection of promising pathways into a coherent translational program.
